# Identification and analysis of long non-coding RNAs in response to H5N1 influenza viruses in duck (*Anas platyrhynchos*)

**DOI:** 10.1186/s12864-018-5422-2

**Published:** 2019-01-11

**Authors:** Chang Lu, Yanling Xing, Han Cai, Yirong Shi, Jinhua Liu, Yinhua Huang

**Affiliations:** 1 State Key Laboratory of Agrobiotechnology, China Agricultural University, Beijing, 100193 China; 2 Key Laboratory of Animal Epidemiology and Zoonosis, Ministry of Agriculture, College of Veterinary Medicine, China Agricultural University, Beijing, China

**Keywords:** Duck, LncRNAs, Avian H5N1 influenza virus, Immune response

## Abstract

**Background:**

Long non-coding RNAs (lncRNAs) are important component of mammalian genomes, where their numbers are even larger than that of protein-coding genes. For example, human *(Homo sapiens)* (96,308 vs. 20,376) and mouse *(Mus musculus)* (87,774 vs. 22,630) have more lncRNA genes than protein-coding genes in the NONCODEv5 database. Recently, mammalian lncRNAs were reported to play critical roles in immune response to influenza A virus infections. Such observation inspired us to identify lncRNAs related to immune response to influenza A virus in duck, which is the most important natural host of influenza A viruses.

**Results:**

We explored features of 62,447 lncRNAs from human, mouse, chicken, zebrafish and elegans, and developed a pipeline to identify lncRNAs using the identified features with transcriptomic data. We then collected 151,970 assembled transcripts from RNA-Seq data of 21 individuals from three tissues and annotated 4094 duck lncRNAs. Comparing to duck protein-coding transcripts, we found that 4094 lncRNAs had smaller number of exons (2.4 *vs.* 10.2) and longer length of transcripts (1903.0 bp *vs.* 1686.9 bp) on average. Among them, 3586 (87.6%) lncRNAs located in intergenic regions and 619 lncRNAs showed differential expression in ducks infected by H5N1 virus when compared to control individuals. 58 lncRNAs were involved into two co-expressional modules related to anti-influenza A virus immune response. Moreover, we confirmed that eight lncRNAs showed remarkably differential expression both in vivo (duck individuals) and in vitro (duck embryo fibroblast cells, DEF cells) after infected with H5N1 viruses, implying they might play important roles in response to influenza A virus infection.

**Conclusions:**

This study presented an example to annotate lncRNA in new species based on model species using transcriptome data. These data and analysis provide information for duck lncRNAs’ function in immune response to influenza A virus.

**Electronic supplementary material:**

The online version of this article (10.1186/s12864-018-5422-2) contains supplementary material, which is available to authorized users.

## Background

Transcriptome analysis has revealed that 80% of eukaryotic (such as human [1] and mouse [2]) genomes are transcribed, but 1–2% of the genome encodes proteins [3], suggesting that a large number of transcripts were non-coding RNAs (ncRNAs). Among ncRNAs, lncRNAs distribute extensively in animal and plant genomes. LncRNAs are transcripts being longer than 200 nucleotides, which were previously reported not to encode functional proteins [4]. However, recent studies found that some lncRNAs (such as DWORF [5] and SPAR [6]) encoded peptides and played important roles in the myocardial contraction and muscle regeneration, respectively. Such observation updated our knowledge about lncRNAs, which may not only affect transcription of gene expression with non-coding transcripts [7, 8], but also encode polypeptides to negatively regulate protein activation with its peptide [9].

Duck is one of the important economical waterfowl and the natural reservoir of all influenza A viruses harboring 18 hemagglutinin (HA) and 11 neuraminidase (NA) subtypes [10], with the exception of the H13, H16, H17 and H18 subtypes [11]. Compared to chicken being destroyed by influenza A virus (such as H5N1 viruses), ducks are tolerant to most subtypes of H5N1 viruses [12]. RIG-I (Retinoic Acid Inducible Gene I), one of the important intracellular viral RNA detector, is absent in chicken but play a critical role in immune response to H5N1 virus infections, partly contributing to the stark difference in influenza pathology between ducks and chickens [13]. Our previous studies indicated that duck might diversify their immune coding genes, such as β-defensin and butyrophilin-like genes, to optimize their immune response to influenza A virus [14]. Recently, lncRNAs were found to modulate influenza viral infection in mammals. For example, *NRAV* inhibits the transcription of interferon-stimulated genes (ISGs) by affecting their histone modification, thus promotes replication of influenza A virus in human and mouse [15]. Similarly, mouse *VIN* promotes the replication of influenza viruses [16]. However, lncRNAs were only characterized on few birds (i.e. chicken and zebra finch) [17, 18] and their functions in anti-influenza immune response in birds are not clear.

Here, we developed a systematical pipeline for lncRNA identification and annotated 4094 duck lncRNAs using brain, lung and spleen transcriptomes of control individuals and ones that were infected with a highly pathogenic (A/duck/Hubei/49/05) or a weakly pathogenic (A/goose/Hubei/65/05) H5N1 virus (DK/49 or GS/65). We compared the genomic structure and expression pattern of duck lncRNAs to their protein-coding genes and identified the differentially expressed lncRNAs in H5N1 virus infected duck and control individuals. We further identified lncRNAs and protein-coding genes co-expressional modules. Moreover, we qualified transcripts of eight lncRNAs by qRT-PCR and found these lncRNAs showing remarkably differential expression in both H5N1 virus infected duck and DEF cells. The findings offer new information for functional studies of duck lncRNAs, especially in immune response to influenza A viruses.

## Results

### Development a pipeline for the identification of lncRNA

**Fig. 1:**
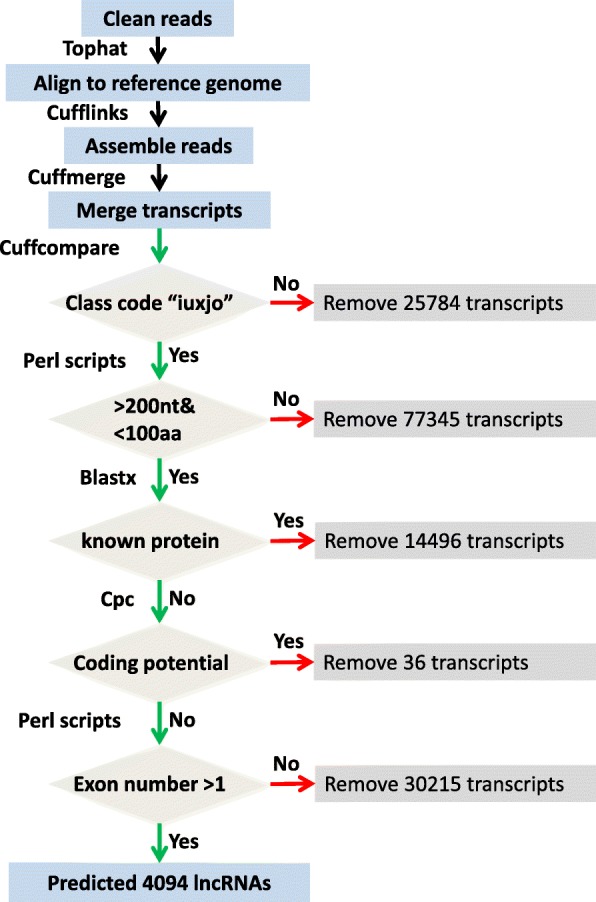
Schematic diagram of the pipeline for identification of duck lncRNAs. Clean reads were mapped and assembled according to the duck assembly using TopHat and Cufflinks. Putative lncRNAs were identified using five criteria: (1) filtered with duck protein-coding genes; (2) length > 200 nucleotides and ORF < 100 amino acids; (3) having no known protein domains; (4) low coding potential (CPC score < 0); (5) more than one exon

A suitable pipeline for the identification of duck lncRNA was developed as the following: (1) explore features of 62,447 lncRNAs of five model organisms (human, *Homo sapiens*; mouse, *Mus musculus*; chicken, *Gallus gallus*; zebrafish, *Danio rerio*; elegans, *Caenorhabditis elegans*) from the GENCODE and NONCODE databases. We found that most lncRNA transcripts are > 200 nucleotides (nt) in length, have more than one exon, and the length of open reading frame (ORF) are < 100 amino acids (AA) (Additional file 1: Figure S1); (2) build a strict platform to identify putative lncRNA transcripts larger than 200 nt and having ORFs shorter than 100 AA according to the features of lncRNA in five model organisms (Fig. 1) [19]; (3) the putative lncRNA is aligned to known protein sequences using BlastX (*E* value < 1 × 10^− 3^) and filtered with the protein database of KEGG (Kyoto Encyclopedia of Genes and Genomes), Swiss-Prot (Swiss-Protein database) and the NR data set from NCBI, as well as duck reference gene set, using the Cuffcompare program from the Cufflinks package [14, 17, 20]; (4) assess the protein-coding potential of putative lncRNAs using the Coding Potential Calculator (CPC) [21]; (5) filter putative lncRNA with only one exon.

### Genome-wide annotation of duck lncRNAs

A total of 2,592,396,390 clean reads from 21 available duck transcriptomes of three tissues (brain, spleen and lung) with or without infection by a highly pathogenic (A/duck/Hubei/49/05) or a weakly pathogenic (A/goose/Hubei/65/05) H5N1 virus after 1, 2 and 3 days were assembled to produce 151,970 transcripts (Additional file 2: Table S1, Fig. 1). We used the above pipeline shown in Fig. 1 to identify duck lncRNAs. Among these assembled transcripts, 126,186 were novel or unknown transcripts and 48,841 were putative lncRNAs having a nucleotide length longer than 200 nt and ORF shorter than 100 AA. We filtered 48,841 transcripts and found 9765 of them being homologous to duck and chicken protein. We further filtered the remained 39,076 transcripts and removed 3731 transcripts being homologous to protein-coding genes in the three protein databases (Swiss-Prot, KEGG and NR). After that, we assessed the protein-coding potential of 35,345 transcripts using the CPC program and deleted 36 potential coding transcripts [21]. Finally, we removed 31,215 transcripts that had only one exon. This effort finally identified 4094 transcripts from 3108 loci as putative multi-exons lncRNAs (Fig. 1 and Additional file 3). In order to identify the accuracy and quality of these annotated duck lncRNAs, we blasted them to the non-redundant database in the NCBI (RefSeq) and found 752 were annotated in this database.

### Characteristics of duck putative lncRNAs

**Fig. 2:**
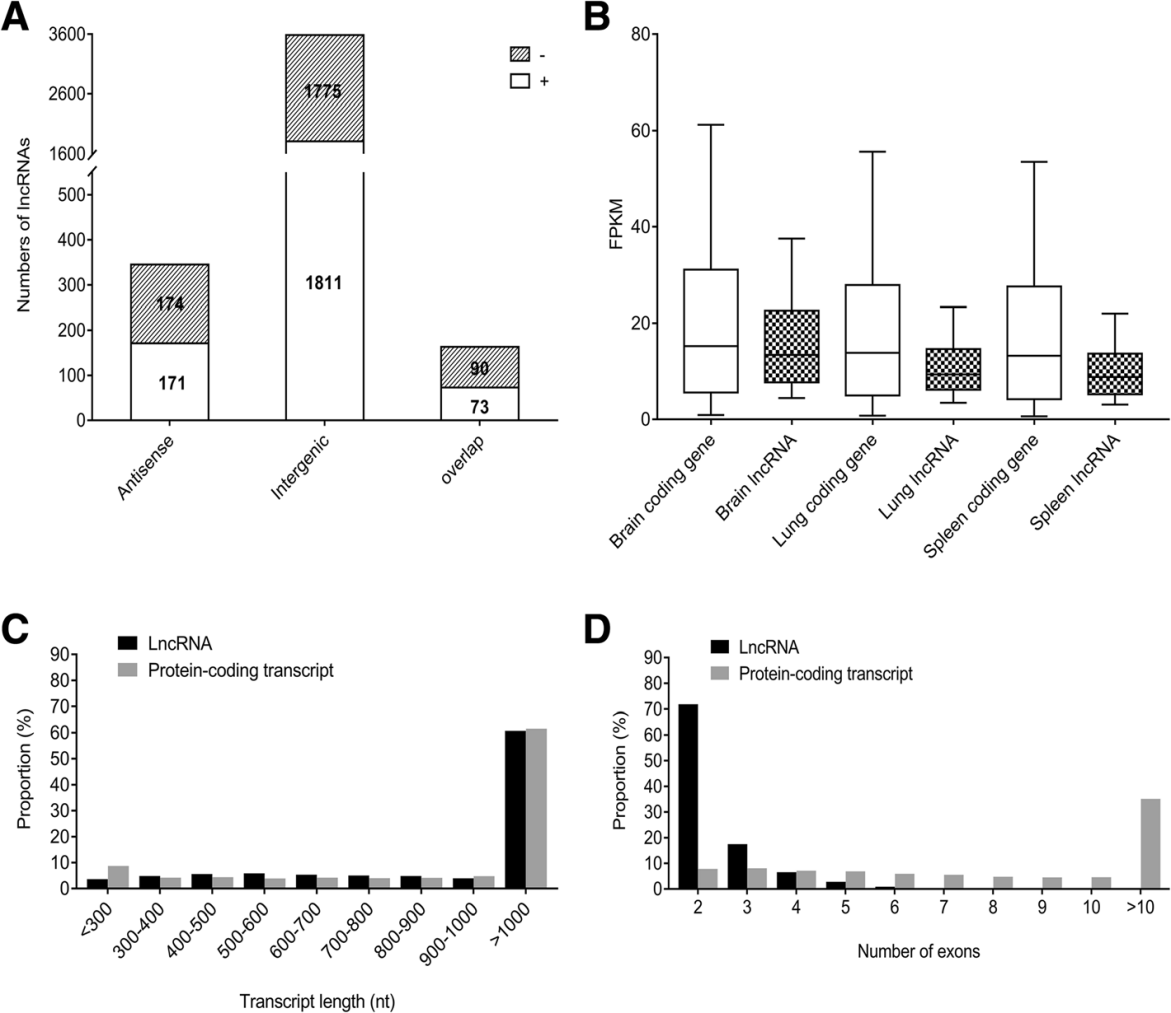
Categories and features of the 4094 predicted duck lncRNAs. **a** Categories of duck lncRNAs divided based on genomic location between lncRNAs and protein-coding genes. “-” or “+” strand for each of the three main types was labeled on the columns. **b-d** Duck lncRNAs are longer, have fewer exons and have lower expression levels than protein-coding genes. Expression (**b**), distribution of length (**c**) and number of exons (**d**) of 4094 lncRNAs and 16,353 protein-coding transcripts of duck (BGI_duck_1.0)

LncRNAs were classified into three types (intergenic, antisense and overlap lncRNAs) according to their locations relative to the nearest protein-coding genes. Among these 4094 duck lncRNAs, a large proportion (87.6%) was located in the intergenic regions, 8.4% was antisense transcripts of protein-coding genes, while only 4.0% was overlapped lncRNAs (Fig. 2a). We further counted the distribution of transcript length, exon number and expression level of 4094 lncRNAs and compared them to 16,353 duck protein-coding transcripts. This effort found that 4094 duck lncRNA transcripts had an average length of 1903 nt, 39.25% of them were 200 to 1000 nt and 60.75% of them were larger than 1000 nt in length (Fig. 2c). In contrast, the average length of duck mRNAs (1687 nt) was smaller than that of duck lncRNAs. For gene structure, duck lncRNAs contained two to nine exons with an average of 2.4 exons. This number is smaller than the average of duck mRNAs (average 10.2) (Fig. 2d). We further compared expressional patterns of lncRNAs and protein-coding genes in three tissues (brain, lung and spleen). The average expression levels of the putative lncRNA genes (brain average 15.7 FPKM, lung average 10.7 FPKM and spleen average 9.9 FPKM) tend to be lower than those of the protein-coding genes (brain average 19.8 FPKM, lung average 17.8 FPKM and spleen average 17.3 FPKM) in all three tissues (Fig. 2b).

### Identification of differentially expressed duck lncRNAs (DElncRNAs)

**Fig. 3:**
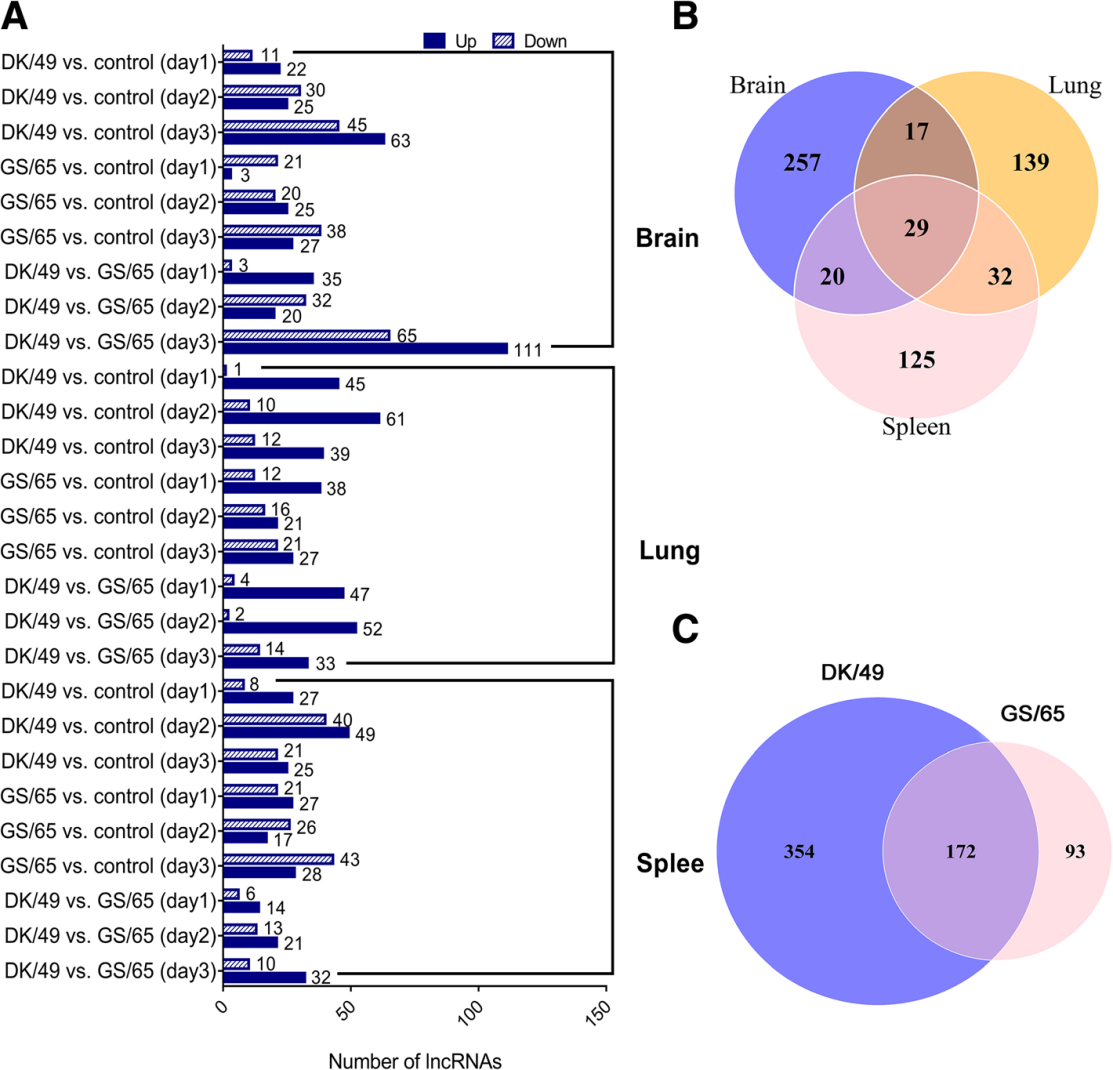
DElncRNAs between infected ducks and control ducks. **a** The numbers of DElncRNAs in three tissues. **b** Venn diagram showing non-overlap and overlap among putative lncRNAs from brain, lung and spleen tissues. **c** Venn diagram showing non-overlap and overlap between putative lncRNAs from DK/49- and GS/65-infected ducks

**Fig. 4:**
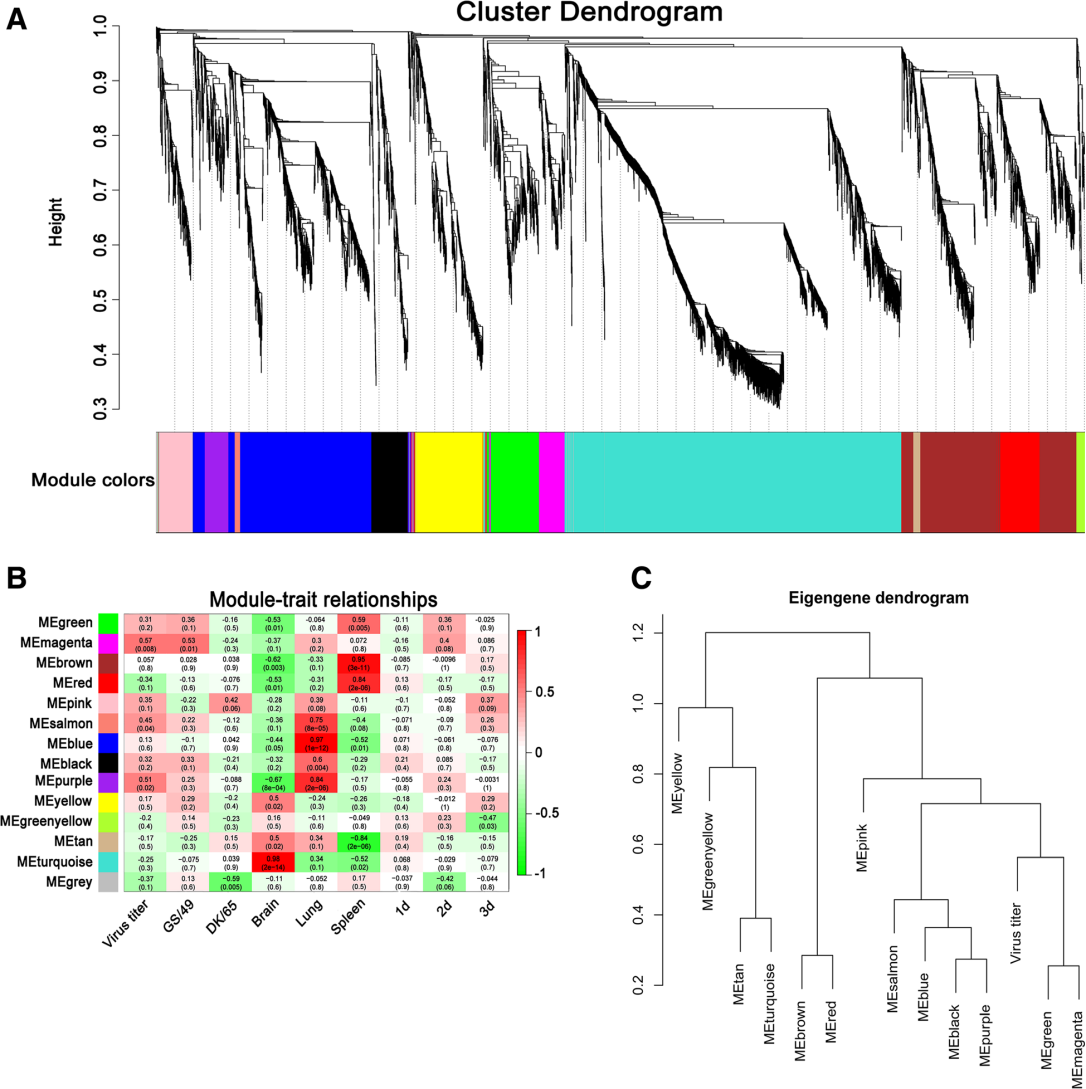
Co-expression network of DElncRNAs and DEGs. **a** Thirteen co-expression modules predicted by WGCNA analysis. **b** Heatmap of correlations between module and virus titer, H5N1 virus strain, tissue and the days. **c** Hierarchical clustering dendrogram modules eigengenes and the RNA-Seq sample trait virus titer

We compared lncRNA expression in duck infected with H5N1 viruses (DK/49-infected or GS/65-infected) against control individuals and identified a total of 619 DElncRNAs (FDR ≤ 0.05, fold change ≥ 2) including 323, 217 and 206 DElncRNAs in the brain, lung and spleen respectively (Fig. 3a and Additional file 4: Table S2). In lung, DK/49-infected ducks had 46, 71 and 51 lncRNAs with significantly changed expression 1–3 d after inoculation, and GS/65-infected ducks had 50, 37 and 48 lncRNAs with significantly changed expression 1–3 d after inoculation when compared to control ducks, while DK/49-infected ducks had 51, 54 and 47 lncRNAs showing significantly differential expression when compared to the corresponding GS/65-infected ducks 1–3 d after inoculation respectively (Fig. 3a and Additional file 4: Table S2). Similar to lung tissue, DK/49- and GS/65-infected ducks showed similar number of (33 *vs.* 24, 35 *vs.* 48) DElncRNAs when compared to control individuals in brain and spleen in early infected time (1 d after inoculation). Such differences in number of DElncRNAs was magnified in later infected time (2–3 d after inoculation), except that DK/49- and GS/65-infected ducks had similar number of DElncRNAs in brain in 2 d after inoculation (Fig. 3a). Detailed analysis indicated that, among 619 duck DElncRNAs, 29 were significantly differentially expressed in all three tissues (Fig. 3b). 172 lncRNAs were differentially expressed in both DK/49-infected and GS/65-infected ducks (Fig. 4c). These results suggested that the DElncRNAs might be involved into immune response to influenza A virus infection in ducks.

### Prediction the target protein-coding gene of lncRNA in *cis* and in *trans*

LncRNAs inhibit or activate the transcription of genes located in their upstream or downstream by recruiting chromatin remodeling factors or protein complex [22]. These lncRNAs function in *cis*, acting on linked genes in the vicinity of the RNA’s site. In order to identify duck lncRNAs functioning in *cis*, we predicted the potential protein-coding gene targets of lncRNAs among differentially expressed genes (DEGs) using 10 kb and 100 kb as the cutoff (Table 1). We detected 972/11,508 lncRNAs-coding genes pairs when used the 10/100 kb as the cutoff, and 721/106 DElncRNAs-DEGs pairs when used the 10/100 kb as the cutoff. We further found that 365 DElncRNAs were neighbored to 627 DEGs within 100 kb, and 86 DElncRNAs were neighbored to 103 DEGs within 10 kb (Table 1 and Additional file 5: Table S3).

**Table 1:** Summary of lncRNA-coding neighbor gene pairs

	lncRNAs	Coding genes	DElncRNAs	DEGs	lncRNA-coding gene pairs	DElncRNA-DEGs pairs
100 kb	2632	7914	365	627	11,508	721
10 kb	747	889	86	103	972	106

We searched for protein-coding genes 100 kb/10 kb upstream and downstream of the identified lncRNAs. lncRNA-coding gene pairs/DElncRNA-DEGs pairs: the number of gene pairs of lncRNAs (or DElncRNAs) and protein-coding genes (or DEGs) by using 100 kb/10 kb as cutoff; 100 kb/10 kb: the number of lncRNAs (or DElncRNAs) and protein-coding genes (or DEGs) by using 100 kb/10 kb as cutoff

Moreover, lncRNAs act in *trans*, regulating genes far away from them or even in other chromosomes [23]. We predicted the targets of lncRNAs in *trans*-regulatory relationships using co-expression analysis [24]. The lncRNAs-coding genes co-expression networks were performed with 619 DElncRNAs and 5594 DEGs using the WGCNA package (Additional file 4: Table S2, Additional file 6: Table S4 and Additional file 7: Table S5). As a consequence, 13 co-expression modules were constructed in size from 2261 genes in the turquoise module to 36 genes in the salmon module (Fig. 4a and Additional file 8: Table S6).

Functional analysis indicated that 5594 expressed DEGs were enriched in 640 GO terms (401 GO under biological process, 144 GO under cellular component and 95 GO under molecular function) (Additional file 9: Table S7). The results showed that DEGs from magenta and green modules were associated with immune response in the biological process, including innate immune response (GO: 0045087), defense response to virus (GO: 0051607), positive regulation of defense response to virus by host (GO: 0002230), immune response (GO: 0006955) and negative regulation of viral genome replication (GO: 0045071).

**Fig. 5:**
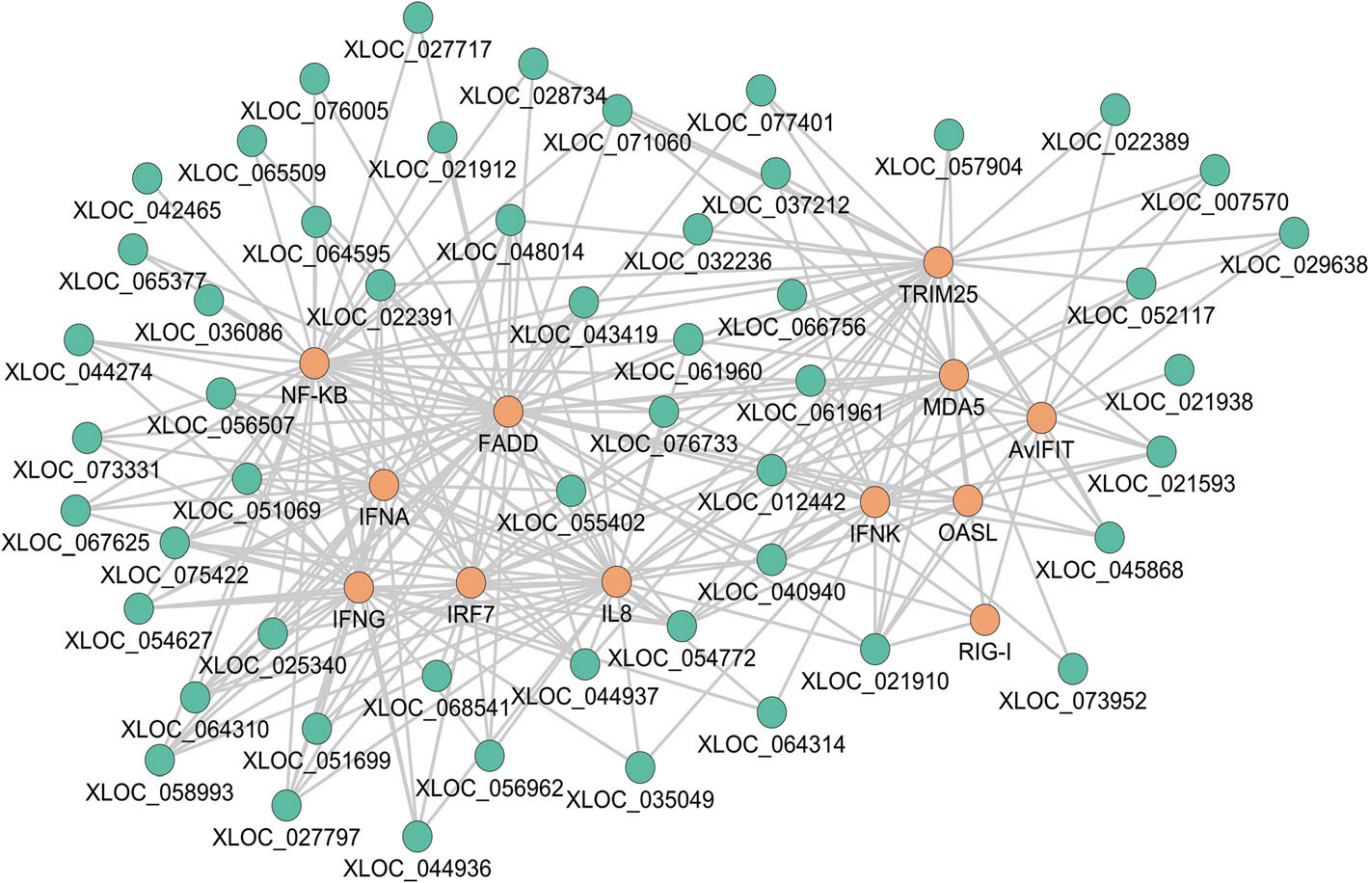
The magenta and green co-expressional networks of DElncRNAs and DEGs. Orange nodes represent lncRNAs, green nodes represent coding genes. The line between lncRNAs and coding genes indicates an expression pattern correlation

Among 13 co-expression modules, magenta module had the highest correlation with virus titer (Fig. 4b, c), which included 16 DElncRNAs and 174 DEGs. Detailed analysis indicated that both the magenta and green modules contained many known anti-influenza A virus immune genes (such as *NF-κB*, *FADD*, *IFNA*, *IFNG*, *IRF7*, *IL8*, *IFNK*, *OASL*, *AvIFIT*, *MDA5* and *TRIM25*) (Fig. 5), supporting their critical role in host response to influenza A virus infection. Such inference was further supported by pathway analysis (KEGG), which demonstrated that DEGs in the magenta and green modules were significantly enriched (*p* < 0.05) in nine pathways including Influenza A, Herpes simplex infection, RIG-I-like receptor signaling pathway, Cytokine-cytokine receptor interaction, Toll-like receptor signaling pathway, NOD-like receptor signaling pathway, Intestinal immune network for IgA production, Arginine biosynthesis, Alanine, aspartate and glutamate metabolism, and Cytosolic DNA-sensing pathway (Additional file 10: Table S8).

### Real-time quantitative PCR (qPCR) validation

**Fig. 6:**
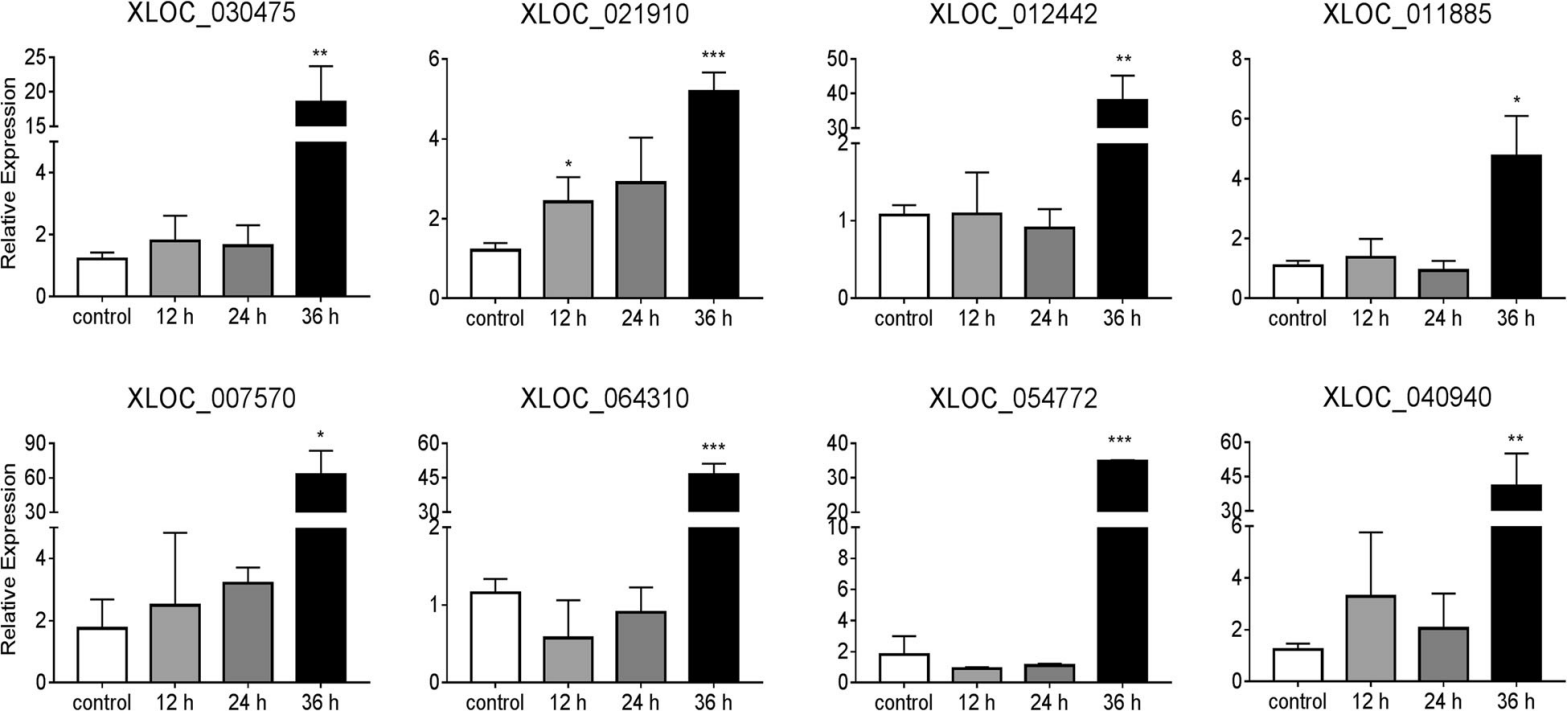
Expression of eight DElncRNAs from magenta and green modules in DEF cells before and after infected with H5N1 virus. Expression in cells were counted relative lncRNA level to that of reference gene─*GAPDH* and presented as fold change versus the corresponding of DEF control cells (without H5N1 virus infection) (two-tailed Student’s test, *n* = 3). The data are expressed as the mean ± SD. *P < 0.05, **P < 0.01, ***P < 0.001

Expression of eight DElncRNAs from the magenta or green modules in DEF cells before or after infected by H5N1 virus (12 h, 24 h and 36 h) were examined using qRT-PCR (Fig. 6). This analysis indicated that, similar to duck in vivo transcriptomic expressional profiles, all tested eight lncRNAs were significantly increased (*p* < 0.05) in DEF cells infected by CK/0513 (A chicken/hubei/0513/2007) H5N1 virus after 36 h. Such consistent expressional distribution of these DElncRNAs in vivo and in vitro was further supported that they might play important roles in host immune response to influenza A virus. Our ability to assess the function of these eight DElncRNAs using genetic manipulations will certainly extend knowledge of lncRNA related to influenza in ducks.

## Discussion

Accumulated studies demonstrated that lncRNAs extensively involved in various biological processes in animals, including embryogenesis [25, 26], muscle development [27, 28], adipogenesis [29] and immune responses [15, 30]. Therefore, it is interested to annotate and identify candidate lncRNA related to diseases, such as influenza, using transcriptomic data. In the present study, we built a screening platform to identify lncRNAs after explored features of 62,447 lncRNAs from five model animals (Fig. 1). This pipeline removed known protein-coding genes using the most effective computational methods available to date, such as Cufflinks and CPC. Unlike the previously reserved intergenic transcripts [31], we retained the intergenic transcripts, antisense transcripts and transcripts that overlap with the protein-coding genes. We removed the transcripts having ORF length more than 100 AA. In addition, we filtered the potential protein-coding genes through blasting with duck protein sequences, chicken protein sequences and other sequences from protein databases (KEGG, Swiss-Prot and NR data sets). This extended range of comparison could remove the transcripts that have encoded potential. Finally, we retained the multi-exons transcripts as predicted lncRNAs according to the characteristics of model animals’ lncRNAs.

LncRNAs are a group of endogenous RNAs that function as regulators of gene expression. We detected 619 putative DElncRNAs among control ducks and individuals infected with H5N1 virus (Fig. 3). We predicted function of DElncRNAs in *cis* and *trans*. The *cis* lncRNAs act on the neighboring genes. In *trans*, gene executes cellular processes in functional modules [32]. Therefore, this study constructed the weighted gene co-expression network and identified modules related to H5N1 virus titer. Among 13 detected modules (Fig. 4b), the magenta module was correlated with virus titer and DK/49 virus, while the other modules were correlated with GS/65 or tissues or the days of duck growth and development after inoculated. Moreover, we found that the green module was highly associated with magenta module and virus titer. Interestingly, *TRIM25*, *IFNK*, *DDX58* (*RIG-I*), *AvIFIT* and *OASL* were found to be a highly connected gene in the magenta module, and *IL8*, *FADD*, *NF-κB*, *IRF7*, *IFNE*, *IFNA* and *CXCL6* in the green module, these protein-coding genes play important roles in anti-virus immune response (Fig. 5). For example, Jiang et al. [33] found an IFN-induced long noncoding RNA lnc-Lsm3b, played a negative feedback regulatory role in the late immune response and inhibited the activity of RIG-I. Lnc-Lsm3b competes to bind RIG-I monomers with viral RNAs using its multivalent long stem domain, prevents conformational changes of RIG-I and activation of its downstream signaling. Here, we found that *RIG-I* was co-expressed with *XLOC_021920*, *XLOC_012442* and *XLOC_040940* (weight > = 0.2) in the magenta and green modules. These three lncRNAs showed differentially expressed in all three H5N1 infections in vivo and in vitro, implying that might affect function of *RIG-I* in the immune response influenza A viruses and be interested to further functional studies.

Due to limitations of available duck transcriptomes, annotations of lncRNAs in this study are not comprehensive. These include: (1) some duck lncRNAs lacking polyadenylation might be not annotated using mRNA transcriptome; (2) due to the inherent limitations of using 90 bp paired-end RNA-Seq, we did not get full sequences of all annotated duck lncRNAs; (3) some lncRNAs may be annotated incorrectly due to error of the available duck assembly.

## Conclusion

LncRNAs have been functionally annotated and studied extensively in mammals. However knowledge even sequences of avian lncRNA, especially duck lncRNA, is scarce. We first identified duck lncRNAs associated with immune response to avian influenza virus using RNA-Seq data. We present a pipeline to investigate the duck lncRNAs and predicted the function of lncRNAs based on the coding gene neighbor loci and the enriched functions of co-expression protein-coding genes. These analyses together with duck lncRNA sequences provide information to understand their functions in the immune response.

## Methods

### Paired-end RNA sequencing

The RNA-Seq data (SRA accession SRP052742) were from the duck genome paper [14].

### Reads mapping and assembly

After clean reads, we build the index of the reference genome [14] using Bowtie v2.2.6 and aligned paired-end clean reads to the reference genome using Tophat v2.1.0. Tophat was run with “-N 3 --read-edit-dist 5 -r 20 -a 9 -i 30 -I 4000 --min-segment-intron 30 -max-segment-intron 4000 --min-coverage-intron 30 --max-coverage-intron 4000 --microexon-search” and “--phred64-quals”, other parameters were set as default. Only the mapped reads with number of mismatches less than three were used to construct transcripts using Cufflinks (v2.2.1) in a reference-based approach. Finally, we used Cuffmerge to merge all the transcripts into a final irredundant transcript GTF file.

### Describing feature of lncRNAs and protein-coding genes

A total of 4094 lncRNAs and 16,353 mRNAs were characterized in transcript length, exon number and expressional profiles. Transcript length categories were < 300, 300–400, 400–500, 500–600, 600–700, 700–800, 800–900, 900–1000, and > 1000 nucleotides. Exon number categories were: 1, 2, 3, 4, 5, 6, 7, 8, 9, 10, and > 10. The proportion of different types of lncRNAs and protein-coding transcripts were calculated. Furthermore, we used brain, lung and spleen transcriptomes of control ducks to characterize the expression pattern of the lncRNA genes and coding genes, whose FPKM value were calculated using the Cufflinks (v2.2.1) program.

### Identifying differentially expressed lncRNAs and neighbor target genes

Differentially expressed lncRNAs between control ducks and ducks infected by H5N1 viruses were identified using the Cuffdiff program with the thresholds of |fold change| ≥ 1 and adjusted *p*-values < 0.05. Protein-coding genes were within 10 kb/100 kb upstream and downstream of the identified lncRNAs were inferred as neighbor target genes.

### Analysis of co-expression, GO enrichment and KEGG pathway

Co-expression networks were built using the WGCNA package [24]. First, we used FPKM value of DEGs and DElncRNAs as the input file (Additional file 7: Table S5). We then chose “soft power = 8” using the scale-free topology criterion. After that, we constructed networks using the blockwiseModules function in the WGCNA package with the minimum module size to 30 genes, and the minimum height for merging modules at 0.15 (default value). For each module, we calculated module membership (also known as module eigengene based connectivity k_ME_) based on Pearson’s correlation [24]. Finally, we used the virus titers of three tissues as trait data to calculate the GS value (gene Trait Significance). We chose modules which had a highly strength connection with virus titers. To visualize the chose modules (together with GO and KEGG analysis), the connections between the lncRNAs and the mRNAs were shown using the Cytoscape program [34].

GO category and KEGG enrichment were performed using DAVID software [35] and KOBAS software [36] using a threshold of *P* < 0.05, respectively.

### Cell culture, RNA isolation and qRT-PCR

Duck embryonic fibroblast (DEF) cells were isolated from eleven-day-old embryos. Duck embryos were digested with trypsin, suspended and filtered using gauze. DEF cells were diluted and cultured in six-well culture plates in an atmosphere of 5% CO_2_ at 37 °C. Cells with a coverage ~ 90% were collected or infected with H5N1 virus. H5N1 virus infected cells were collected after infection at 12, 24 and 36 h, respectively.

Total RNA was isolated from cells using RNeasy® Mini Kit (QIAGEN, Germany). RNA quality was measured using a NanoDrop-2000 spectrophotometer (Thermo Fisher Scientific, USA) and by agarose gel electrophoresis. Genomic DNA was removed from extracted total RNA using DNase treatment. A 2 μg aliquot of total RNA was used for cDNA synthesis using a M-MLV Reverse Transcriptase (Promega, USA) with Oligo (dT)_15_ Primer.

Quantitative RT-PCR was performed using SYBR Green PCR Master Mix with a real-time PCR System ABI7500 (ABI, USA). Quantitative RT-PCR condition were as follows: 94 °C for 2 min, followed by 40 cycles of 95 °C for 5 s and 60 °C for 30s. Fluorescence changes of SYBR Green were monitored automatically in each cycle, and the threshold cycle (Ct) over the background was calculated for each reaction. Samples with three biological replicates were normalized using *GAPDH*, and the relative expression levels were measured using the 2^-△△Ct^ analysis method. Student’s *t*-test was used to determine whether the qRT-PCR results were statistically different from two samples (*P < 0.05; **P < 0.01). PCR primers are listed in Additional file 11: Table S9.

## Additional files

Additional file 1: **Figure S1.** Features of lncRNA in five organisms. Length distribution of duck lncRNAs transcript (A), ORF length (B) and numbers of exons (C) were shown in the picture. (TIF 3391 kb)

Additional file 2: **Table S1.** Summary of RNA-Seq data and reads mapped to the *Anas platyrhynchos* genome. (DOCX 21 kb)

Additional file 3: Information of lncRNA annotated with GTF files. (GTF 1785 kb)

Additional file 4: **Table S2.** DElncRNAs detected between duck infected with DK/49 or GS/65 H5N1 virus and control individuals. (XLSX 176 kb)

Additional file 5: **Table S3.** Protein-coding genes located within 10/100 kb upstream and downstream of duck lncRNAs. (XLSX 642 kb)

Additional file 6: **Table S4.** List of DEGs used in co-expression analysis. (XLSX 2055 kb)

Additional file 7: **Table S5.** FPKM value of DElncRNAs and DEGs.(CSV 1273 kb)

Additional file 8: **Table S6.** Thirteen co-expression modules were predicted with the WGCNA package. (CSV 2346 kb)

Additional file 9: **Table S7.** GO enrichment analysis of DEGs co-expressed with DElncRNAs. (XLSX 135 kb)

Additional file 10: **Table S8.** KEGG analysis of DEGs co-expressed with DElncRNAs. (XLSX 83 kb)

Additional file 11: **Table S9.** Primers used in qRT-PCR analysis. (DOCX 18 kb)

## Abbreviations

DEGs: Differentially expressed genes
DElncRNAs: Differentially expressed lncRNAs
DK/49: A highly pathogenic (A/duck/Hubei/49/05) H5N1 virus
GO: Gene ontology
GS/65: A weakly pathogenic (A/goose/Hubei/65/05) H5N1 virus
KEGG: Kyoto Encyclopedia of Genes and Genomes
LncRNAs: Long non-coding RNAs
ORF: Open reading frame
