# Supplementary figures and images for: Identification and analysis of long non-coding RNAs in response to H5N1 influenza viruses in duck (Anas platyrhynchos)
Source: BMC Genomics. 2019 Jan 11;20:36. doi: 10.1186/s12864-018-5422-2 (PMC6330444; doi:10.1186/s12864-018-5422-2)

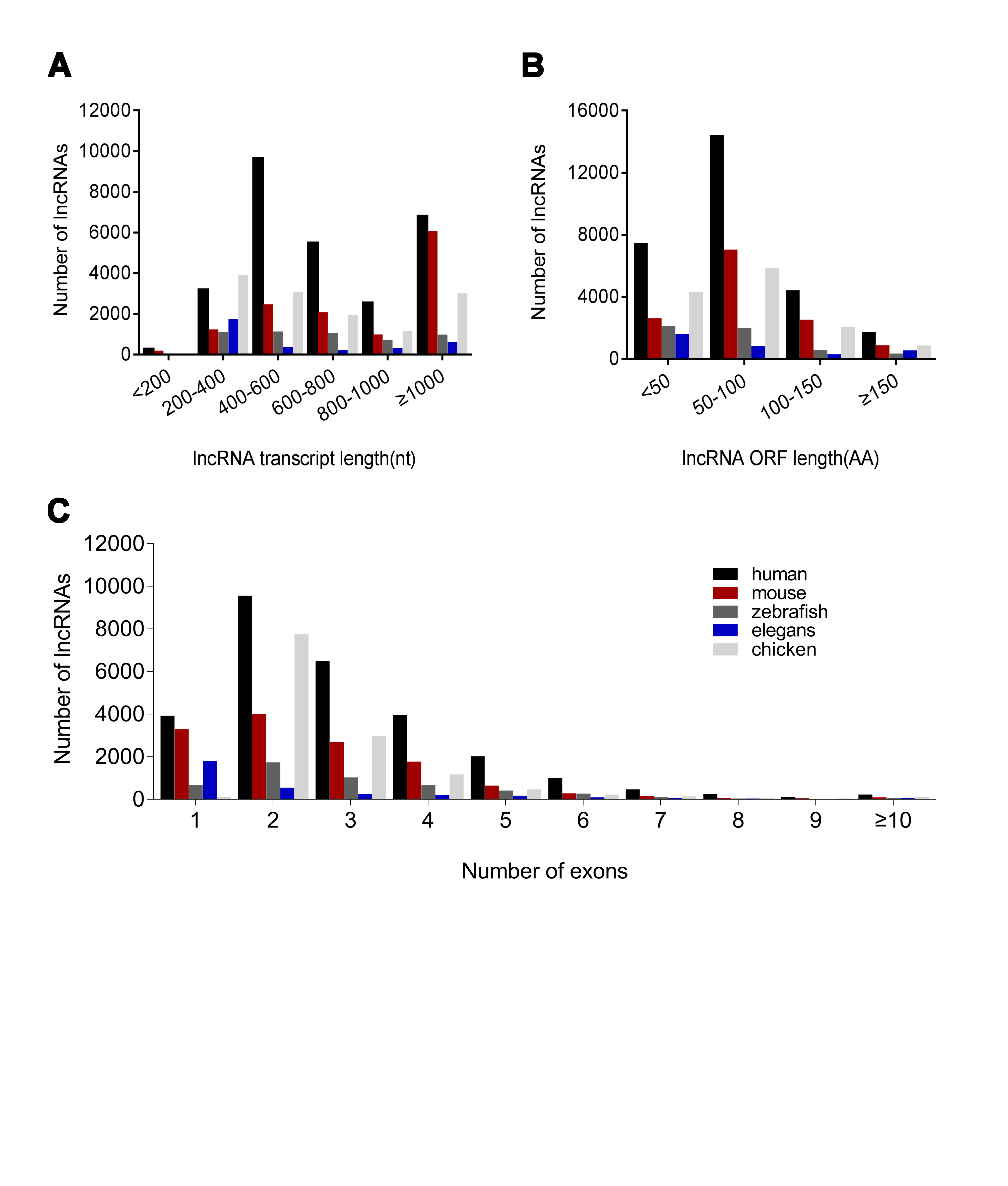

Supplement: Supplementary file 1 — Figure S1. Features of lncRNA in five organisms. Length distribution of duck lncRNAs transcript (A), ORF length (B) and numbers of exons (C) were shown in the picture. (TIF 3391 kb) [file 12864_2018_5422_MOESM1_ESM.tif]
