# Supplementary material for: Identification and analysis of long non-coding RNAs in response to H5N1 influenza viruses in duck (Anas platyrhynchos)
Source: BMC Genomics. 2019 Jan 11;20:36. doi: 10.1186/s12864-018-5422-2 (PMC6330444; doi:10.1186/s12864-018-5422-2)
Supplement: Supplementary file 2 — Table S1. Summary of RNA-Seq data and reads mapped to the Anas platyrhynchos genome. (DOCX 21 kb) [file 12864_2018_5422_MOESM2_ESM.docx]

**Table S1. Read count statistics of the Illumina RNA sequencing data for the 21 libraries studied.**

|  |  | **Raw data** |  | **Clean data** | |  | **Mapped reads** | | | |
| --- | --- | --- | --- | --- | --- | --- | --- | --- | --- | --- |
| **Library** | **Type** | **Total reads** |  | **Total reads** | **Rate (%)** |  | **Left reads** | **Right reads** | **Total reads** | **Rate (%)** |
| Control_brain | 90nt,PE | 138,109,868 |  | 124,941,780 | 90.5 |  | 41,066,536 | 40,816,469 | 81,883,005 | 65.5 |
| HuB49_brain_1d | 90nt,PE | 167,075,304 |  | 150,273,624 | 89.9 |  | 50,198,593 | 50,105,894 | 100,304,487 | 66.7 |
| HuB49_brain_2d | 90nt,PE | 136,581,174 |  | 120,753,246 | 88.4 |  | 41,165,063 | 41,046,840 | 82,211,903 | 68.1 |
| HuB49_brain_3d | 90nt,PE | 127,327,020 |  | 113,431,320 | 89.1 |  | 40,916,643 | 40,807,527 | 81,724,170 | 72.0 |
| HuB65_brain_1d | 90nt,PE | 127,917,046 |  | 117,314,540 | 91.7 |  | 36,806,231 | 36,766,173 | 73,572,404 | 62.7 |
| HuB65_brain_2d | 90nt,PE | 103,925,962 |  | 97,124,064 | 93.5 |  | 32,833,834 | 32,795,078 | 65,628,912 | 67.6 |
| HuB65_brain_3d | 90nt,PE | 141,542,250 |  | 129,675,518 | 91.6 |  | 44,315,817 | 44,250,759 | 88,566,576 | 68.3 |
| Control_lung | 90nt,PE | 152,301,566 |  | 138,589,318 | 91.0 |  | 51,217,720 | 50,915,394 | 102,133,114 | 73.7 |
| HuB49_lung_1d | 90nt,PE | 120,722,698 |  | 109,432,620 | 90.6 |  | 36,140,349 | 36,086,764 | 72,227,113 | 66.0 |
| HuB49_lung_2d | 90nt,PE | 138,225,576 |  | 122,314,850 | 88.5 |  | 42,894,136 | 42,748,854 | 85,642,990 | 70.0 |
| HuB49_lung_3d | 90nt,PE | 140,945,832 |  | 126,129,206 | 89.5 |  | 47,278,804 | 47,132,743 | 94,411,547 | 74.9 |
| HuB65_lung_1d | 90nt,PE | 142,536,150 |  | 130,528,082 | 91.6 |  | 48,577,853 | 48,474,042 | 97,051,895 | 74.4 |
| HuB65_lung_2d | 90nt,PE | 130,926,454 |  | 122,131,996 | 93.3 |  | 43,716,347 | 43,651,802 | 87,368,149 | 71.5 |
| HuB65_lung_3d | 90nt,PE | 118,590,244 |  | 108,694,846 | 91.7 |  | 41,058,725 | 41,001,961 | 82,060,686 | 75.5 |
| Control_spleen | 90nt,PE | 126,777,602 |  | 114,869,132 | 90.6 |  | 40,702,112 | 40,454,673 | 81,156,785 | 70.7 |
| HuB49_spleen_1d | 90nt,PE | 143,544,182 |  | 128,656,816 | 89.6 |  | 44,058,118 | 43,994,183 | 88,052,301 | 68.4 |
| HuB49_spleen_2d | 90nt,PE | 142,242,984 |  | 125,466,658 | 88.2 |  | 43,554,502 | 43,428,852 | 86,983,354 | 69.3 |
| HuB49_spleen_3d | 90nt,PE | 139,735,856 |  | 124,606,014 | 89.2 |  | 44,140,773 | 44,012,035 | 88,152,808 | 70.7 |
| HuB65_spleen_1d | 90nt,PE | 146,395,336 |  | 133,897,730 | 91.5 |  | 47,197,218 | 47,132,575 | 94,329,793 | 70.4 |
| HuB65_spleen_2d | 90nt,PE | 133,839,220 |  | 123,185,378 | 92.0 |  | 43,514,841 | 43,458,095 | 86,972,936 | 70.6 |
| HuB65_spleen_3d | 90nt,PE | 143,559,326 |  | 130,379,652 | 90.8 |  | 44,034,791 | 43,995,278 | 88,030,069 | 67.5 |
| **all sample reads** |  | 2,862,821,650 |  | 2,592,396,390 |  |  | 905,389,006 | 903,075,991 | 1,808,464,997 |  |

HuB49: infected with a highly pathogenic (A/duck/Hubei/49/05) H5N1 virus;

HuB65: infected with a weakly pathogenic (A/goose/Hubei/65/05) H5N1 virus;

PE: paired-end; 1d, 2d, 3d: infected with H5N1 virus 1day, 2days and 3 days.
