# Supplementary material for: Identification and analysis of long non-coding RNAs in response to H5N1 influenza viruses in duck (Anas platyrhynchos)
Source: BMC Genomics. 2019 Jan 11;20:36. doi: 10.1186/s12864-018-5422-2 (PMC6330444; doi:10.1186/s12864-018-5422-2)
Supplement: Supplementary file 11 — Table S9. Primers used in qRT-PCR analysis. (DOCX 18 kb) [file 12864_2018_5422_MOESM11_ESM.docx]

Table S9. Primer pair sequences used in Quantitative PCR.

| **Duck lncRNA name** | **Forward (F) or Reverse (R)** | **Sequence (5’-3’)** |
| --- | --- | --- |
| XLOC_030475 | F | ATTTCTCATCCCTTCAGGTTCAG |
|  | R | TGTTAGTCATCAGAGTGGGTGCT |
| XLOC_021910 | F | CCCCACCACAGGAGACCAAT |
|  | R | TGGGAGATGGGAAGGTAGTTTGT |
| XLOC_012442 | F | AGCAGAAAAGGTTGGCATCG |
|  | R | AGGAAACAAGTTAGAAGGGAGGAG |
| XLOC_011885 | F | GGCAGTTCTAGGGAGACGACA |
|  | R | CACAGTGATGGCTGGCAAA |
| XLOC_007570 | F | GCTTGGTTTCACTTTCTGGTTCC |
|  | R | TGTTGTTGTGGCTTGCCTTTTA |
| XLOC_064310 | F | CACTCCATAAATATCCCCACCT |
|  | R | TGGTCATAATGCAATCCCCAA |
| XLOC_054772 | F | TTCGGATTTAACCATTCAAGGC |
|  | R | CCCATCGGGGTATGTGGAGTA |
| XLOC_040940 | F | AGGGCTGGAAATAATCAAGGC |
|  | R | GGTTCAGTTACTCCAAGTGCCA |
